# Antioxidants and Skin Protection

**DOI:** 10.3390/antiox9080704

**Published:** 2020-08-04

**Authors:** María Herranz-López, Enrique Barrajón-Catalán

**Affiliations:** Instituto de Biología Molecular y Celular (IBMC) and Instituto de Investigación, Desarrollo e Innovación en Biotecnología Sanitaria de Elche (IDiBE), Universitas Miguel Hernández (UMH), 03202 Elche, Spain; mherranz@umh.es

Natural products have a long history of use for skincare and the improvement of the appearance and function of aged and/or damaged skin. Among them, bioactive peptides, oligosaccharides, plant polyphenols, carotenoids, vitamins, and polyunsaturated fatty acids are the most widely used ingredients. 

In recent decades, natural products have undergone rigorous testing, resulting in the identification of phytochemical compounds such as antioxidants with important potentials for the development of cosmetics, cosmeceuticals, and nutraceuticals. Supplementation with these products has been shown to have an effect on the signs of ageing in several human trials.

In this Special Issue, up to 12 original manuscripts and a review are included, covering most of the topics related to the use of natural compounds for skincare and protection. This editorial aims to highlight the most relevant contributions of each single manuscript to the current “state of the art” and provide global significance for the use of antioxidants in skincare. 

Agulló-Chazarra and co-workers [1] have investigated the use of an agriculture by-product to obtain potential ingredients for the cosmetic industry. They have used sweet cherry stems, a novel by-product that has hardly been explored until now. In addition, they have developed an interesting approach, using a multistep procedure covering several aspects of antioxidant and related bioactive properties to select the best extract and propose it as an interesting ingredient for the cosmetic industry. Benincasa et al. [2] have used another raw material to develop their study; in this case, they have studied the use of olive oil by-products to obtain a pure compound, hydroxytyrosyl oleate (HtyOle), and then study its antioxidant and regenerative properties on human keratinocytes. The use of by-products from agriculture is really interesting, because it aligns with the Sustainable Development Goals of the United Nations for 2030 [3] and promotes the use and valorisation of non-previously or low-used materials for high-value applications. 

The photoprotective properties of natural products has been addressed by five of the original articles of this Special Issue as their central topic [4,5,6,7,8]. These articles show up the relevance of photoprotection not only in cosmetics but also in cancer research, as UV radiation is the main cause of some melanoma subtypes. In the first of these articles, Kuo et al. [4] focused on the role of *N*-(4-bromophenethyl)-caffeamide (K36H), a propolis derivative, as a protective compound against UVA-induced apoptosis. This compound has showed its antioxidant activity by reducing reactive oxygen species (ROS) generation and decreasing metalloproteinase expression, DNA damage, and inflammation. The authors has also identified the main molecular mediators of all these actions and provided a global mechanism of how K36H acts inside human keratinocyte cells. A similar approach has been used by Wu et al. in their contribution to this Special Issue [8], testing the photoprotective and anti-inflammatory properties of a sesamin derivative on human fibroblasts.

Tavares and colleagues has used mouse fibroblasts, human keratinocytes, and full-thickness reconstructed human skin to demonstrate the photoprotective actions of fucoxanthin [7]. Their results show that this compound is able to reduce ROS even when used in a prototype of final formulation onto reconstructed skin models. They have also included some studies regarding fucoxanthin’s phototoxicity, concluding that when administered onto reconstructed skin, there are no negative effects, suggesting the possibility of fucoxanthin to become a new photoprotective ingredient for future formulations.

Microvasculature is often forgotten when studying skin, but this Special Issue includes an original manuscript addressing this topic. In this manuscript [5], Noza and colleagues have studied the photoprotective action of a *Rhus coriaria* L. dried fruit extract on microvascular endothelial cells (HMEC-1), finding that the antioxidant activity of this extract is critical for its photoprotective activity and pointing out the relevance of this cellular model to skin studies.

Sanchez-Marzo et al. [6] studied the photoprotective action of two complex formulations containing different combinations of citrus and olive extracts and rosemary diterpenes. They have included ROS accumulation, DNA damage, and photoprotective studies. This study is especially relevant as it tries to correlate the composition of each formulation with its biological effects, increasing the knowledge about the biological action of these compounds included in complex formulations. In fact, the use of these complex products is very interesting for the cosmetic industry, which is continuously looking for new and innovative products involving different activities. For this purpose, natural extracts, including a complex mixture of natural compounds and covering different cosmetics activities, are really good candidates. However, a good analytical and biological characterization of these extracts is crucial to them to become really interesting ingredients for the cosmetic industry, and some good examples can be extracted from this Special Issue.

Another two original articles of this Special Issue include the study of the relationship between antioxidant compounds and melanin production, with the aim of characterising new whitening ingredients from germinated riceberry rice extract [9] and sesamol [10]. This kind of ingredient is interesting not only from a cosmetic point of view but also can be very useful in diseases that induce skin hyperpigmentation, such as melasma or Addison’s disease, or even normal physiological conditions such as pregnancy.

The antipollution effect of antioxidant natural products is also covered in this Special Issue by the work of Moon et al. [11]. In this study, the authors use microwave-assisted extraction to obtain an *Opuntia humifusa* extract able to reduce the harmful effects of particulate matter collected from subway stations. Although the cosmetic consequences of environmental pollution are not the most important ones, it is well known that the skin suffers relevant and significative alterations when exposed to contaminant solid particles, so the need of new antipollution agents is an emerging opportunity for cosmetic ingredient development.

The clinical use of antioxidants for promoting skin health is addressed in this Special Issue by Chaouni et al. [12]. These authors have developed a pilot study on humans about the relationship of antioxidant markers with the skin side effects of radiotherapy. Although this study needs to be expanded, it establishes the basis for the future use of these markers in clinical practice as predictive markers. 

Couto et al. [13] present in this Special Issue a more generalist study about the antioxidant properties of strawberry extract and its putative use as a cosmetic ingredient. They have developed different assays, including a whole extract characterization, which is fundamental, as mentioned above, for any single extract to be really considered as an interesting cosmetic ingredient. These authors also include a final formulation study, in which they have incorporated their extract into a gel and studied its physicochemical properties, stability, cytotoxicity, and ROS scavenging activity. This last aspect is not irrelevant, as there are so many ingredients that are not able to maintain their biological activities when incorporated in a final cosmetic formulation. In this sense, it is recommendable to include final formulation experiments when developing new ingredients, as galenic and pharmaceutical technology is one of the most important aspects to be covered during new product development.

Nutraceutics are also present in this Special Issue, being covered by the review authored by Aguirre-Cruz et al. [14]. This review addresses the use of collagen hydrolysates for skin protection and compares oral and topical administration routes. As collagen hydrolysates are used worldwide to treat joint disorders, this review is really worthy, as the authors have tried to cover all the available information about the use of these products. 

Finally, as a concluding remark, this Special Issue has tried to give a global point of view of how the research on natural compounds to treat skin health is going, but further studies are still needed to increase the scientific evidence not only of new compounds or ingredients, but also of previously described ones with lower scientific support. In this sense, the use of natural compounds should not be only a mere fashion or trending topic, but a new way to discover new and better ingredients to improve skin health.
